# U.S. Primary Care Practice Capabilities Linked to Language Services for Patients with Limited English Proficiency

**DOI:** 10.1007/s11606-025-09968-8

**Published:** 2025-12-01

**Authors:** Stacy Chen, Jenny S. Guadamuz, Hector P. Rodriguez, Karen E. Schifferdecker

**Affiliations:** 1 Division of Health Policy and Management, School of Public Health, University of California, Berkeley, USA; 2 The Dartmouth Institute for Health Policy and Clinical Practice, Geisel School of Medicine, Dartmouth College, Lebanon, NH, USA

**Keywords:** primary care, organizational behavior, immigrant health, language services, ownership

## Abstract

**BACKGROUND:**

Patients with limited English proficiency (LEP) experience more challenges with clinician communication than English-proficient patients. U.S. federal policies require the provision of language services, but little is known about language service availability in adult primary care practices nationally.

**OBJECTIVE:**

To identify primary care physician practice capabilities associated with the routine availability of language services for patients with LEP.

**DESIGN:**

Nationally representative physician practice survey data from the National Survey of Healthcare Organizations and Systems were linked to IQVIA OneKey data and the American Community Survey (*n* = 1226). Multivariable logistic regression models were estimated to assess the association of practice characteristics with availability of language services.

**MAIN MEASURES:**

Whether a practice “always” provides professional language services.

**KEY RESULTS:**

Fifty-four percent of U.S. primary care practices always provide language services. In adjusted analyses, practices participating in an ACO (odds ratio (OR) = 2.21, *p* < 0.01), owned by a healthcare system or hospital (OR = 6.0, *p* < 0.01), or with FQHC status (OR = 3.10, *p* < 0.01) were more likely to provide language services than non-ACO, non-FQHC, or independently owned practices. Practices with relatively high revenue from commercial health insurance (OR = 0.76, *p* < 0.01) were less likely to provide language services, whereas practices with relatively high Medicaid revenue (OR = 1.33, *p* < 0.01) were more likely.

**CONCLUSIONS:**

Even though it is required by law, almost half of U.S. primary care practices do not always provide language services to patients with LEP. Independently owned practices are less likely to offer language services than those owned by healthcare systems or hospitals, or practices with FQHC status, suggesting practice ownership can influence availability. ACO participation and a higher payer mix of Medicaid revenue are associated with language services availability, highlighting that policy reforms can support the routine provision of language services at the practice-level.

## INTRODUCTION

Approximately 25 million people, or 8% of the U.S. adult population, speak English less than very well, also known as having limited English proficiency (LEP). Most (70%) of these adults report having faced language barriers when accessing healthcare services.^1,2^ Adults with LEP often have lower health literacy compared to fluent English speakers, increasing the risk of adverse health outcomes due to language barriers between clinicians and patients.^3–8^ Language barriers can hinder individuals from fully obtaining, processing, and understanding health information, impeding access to care.^3,7,9^ To address these challenges, healthcare services must be provided in the patient’s preferred language, either through offering language services including interpreters and translated written materials, or matching patients with a clinician who has certified language skills.^10^

A qualified medical interpreter provides real-time interpretation between clinicians and patients, incorporating cultural terms, expressions, and idioms to ensure culturally responsive care.^6^ Care delivered by qualified interpreters or language concordant clinicians is associated with improved outcomes, reduced wasteful medical spending, and fewer errors for patients with LEP.^7,9,11^ However, most studies rely on survey-based information about the availability of interpreters at the practice or system level, and rarely specify the interpreter’s modality (i.e., in-person, telephone, or video) and training/certification requirements, which limits inferences about service quality. The U.S. lacks a single, federally mandated certification standard.^6^ In practice, there are several certification pathways which typically require over 40 hours of healthcare interpreter training and exams. Additionally, states and payers have varying accreditation requirements. This variability and lack of consistent measurement make it difficult to understand service quality.

Federal regulations, such as the Civil Rights Act and the Affordable Care Act, require health care organizations to provide language services for patients at no cost if their medical providers receive federal funding.^6,10,12,13^ Despite compelling evidence supporting improved patient outcomes and regulatory mandates, only a limited number of healthcare practices meet language needs.^14–16^ According to the Centers for Medicare and Medicaid Services (CMS), a significant portion of clinicians rely on informal interpreters from family and friends (38%), and almost a third lack a comprehensive language service plan.^6,17^ A study of non-federally funded safety-net clinics found that only 18% utilized professional in-person language services.^17^

Primary care practices are central to ensuring the provision of evidence-based preventative care and early care management.^18^ However, they can encounter barriers to integrating interpreter services into routine practice, including limited care management capabilities, challenges associated with practice ownership, and practice finances. In terms of limited care capabilities, practices often experience challenges with efficiently scheduling interpreter services or pairing patients with available clinicians who speak their language proficiently.^14^ Practice ownership can also influence the routine provision of language services, as practices under different ownership may have varying administrative support, resource availability, management practices, and financial pressures.^19–21^

In terms of practice finances, high personnel costs and a lack of comprehensive payment are significant barriers to utilizing qualified medical interpreters.^16^ In 2020, interpreter services range from $45 to $150 per hour for in-person interpreters and $75 to $210 per hour for phone/video interpreters.^16,22^ Despite high costs of integrating an interpreter workforce, payers such as Medicare and many private insurers do not have a standard requirement to pay practices for language services.^13^ Practices then must incorporate the cost of these services into the regular payment rate for the associated direct service, much of which fall under medical assistance–related or administrative expenditures. Practices serving patients with Medicaid or Children’s Health Insurance Program (CHIP) coverage may receive reimbursement for interpreter services as administrative expenses at a federal matching rate of 50–90%, depending on the state.^23^ Medicaid payment per office visit can be as low as $12, meaning that providing interpreter services would result in financial losses because interpreter services are more costly than payment for the visit.^24^ Consequently, many practices cover language services’ expenses from philanthropy, grants, and reserves without adequate payment. Practices can also agree to alternative payment model contracts, such as Accountable Care Organizations (ACOs), which incentivize quality improvement and management of costs under a global budget with performance-based financial incentives.^25^ However, the extent to which ACO participation encourages provision of language services remains unclear.

Given the importance of language services in primary care and existing federal regulations, evidence about language services availability and practice-level factors associated with its availability is needed. No national evidence, however, exists to characterize primary care practice language services availability. This study aims to quantify practice adoption of language services and identify practice characteristics associated with the provision of these services for patients with LEP. The long-term goal is to inform researchers and policymakers of strategies to improve organizational adoption and implementation of language services for patient populations with LEP.

## METHODS

### Data Sources

We used linked data from several sources: the National Survey of Healthcare Organizations and Systems (NSHOS II), IQVIA OneKey database, and the American Community Survey (ACS). NSHOS II is a cross-sectional, nationally representative survey of physician practices collected using a two-stage sampling design with multi-layered clustering and stratification by organization type.^26^ The sample included practices identified from 2020 IQVIA data with three or more primary care physicians (PCP). IQVIA data, which leverages the American Medical Association’s physician Masterfile, describe relationships among physicians, practices, hospitals, and healthcare systems.^21,27^ We surveyed a cluster stratified random sample of adult primary care physician practices, defined as sites with three or more primary care clinicians at the same location, stratified based on practice ownership and Federally Qualified Health Center (FQHC) status (excluding pediatric practices). Primary care physicians included family medicine, geriatrics, internal medicine, and preventive medicine specialties. Practices also include advanced practice clinicians, such as nurse practitioners and physician’s assistants (Table 1).

Executive leaders or practice managers for each selected practice were surveyed between June 2022 and February 2023. Surveys were administered by SSRS (a market research firm) through mailed outreach, including a notification letter, survey packet with a $10 incentive and online link, a follow-up packet if needed, and an additional $40 incentive upon survey completion. Practices with > 50% missing survey items were excluded. Practice characteristics from the 2022 IQVIA OneKey database were linked to NSHOS II responses along with geographically linked county-level information from the ACS. The ACS provided county-level characteristics, including the percentage of the population who speak English less than very well and the different languages spoken among this population. Of the 1244 survey responses, 1226 were included in the analytic sample after excluding non-responses with missing data for key study variables such as the outcome of interest and ownership status. Survey weights reflected the probability of a practice’s inclusion in NSHOS Wave 1 or 2022 sampling frames and adjusted for non-response, allowing the analytic sample to generalize to the 2022 population of U.S. adult primary care practices with three or more physicians.^21^

### Measures

#### Outcome Measure.

The study outcome was the availability of language services based on the question, “How often is your practice able to provide professional language services for patients with limited English proficiency?” This variable was dichotomized and defined as “yes” if services are “always” provided and “no” if the services were reported to be “often,” “sometimes,” or “never” provided. This measure does not capture the modality of interpretation.

### Main Independent Variables

We assessed three types of practice characteristics: finances, ownership, and organizational capabilities.

#### Finances.

Payer mix was defined as a percentage of total revenue among commercial insurance, Medicare, Medicaid, and uninsured/self-pay. These were ordinal variables categorized as (1) 0%, (2) < 10%, (3) 10–19%, (4) 20–49%, (5) 50–79%, and (6) > 80%. Second, ACO participation or capitated payments indicated whether a practice participated in a commercial capitated contract or ACO contracts with any payer. Third, physician compensation incentives were defined as whether or not any proportion of physician compensation was tied to patient experiences or satisfaction.

#### Ownership.

Practice ownership was categorized as independently owned, medical group, hospital or healthcare system, or federally qualified health center (FQHC) status.

#### Organizational Capabilities.

Organizational capabilities were assessed using two composite measures. Cronbach’s alpha (α) was used to assess the internal consistency of composite measures, with values above 0.70 indicating acceptable reliability.^28,29^ Based on exploratory factor analysis, we developed two composite scales: (1) staff training (α = 0.84) and (2) care processes for complex, high-need patients (α = 0.91). Staff training is a 5-item scale that includes staff training to care for minoritized racial and ethnic populations, staff training to care for immigrant populations, clinician training to care for minoritized racial and ethnic populations, clinician training to care for immigrant populations, and collecting patient preferred language. Care processes for complex, high-need patients is a 5-item scale that includes systems to identify complex, high-need patients, non-physician involvement in care coordination, non-physician involvement in adhering to care plans, non-physician involvement in supporting health risk modification, and non-physician involvement in supporting medication adherence. Responses were dichotomized (no = 0, yes = 1) and a composite score was calculated by averaging the mean responses among the five items, which ranged from 0 to 1. We examined correlations among the survey composite measures and found that none were highly correlated (*r* < 0.70), indicating that they capture distinct practice characteristics (eTable 1).

### Control Variables

To account for need-based factors, we controlled for language services needs at the county-level: measured as the percent of the population aged 5 + who speak English less than “very well”: < 5% (low need), 5–10% (medium need), and > 10% (high need), which is relevant because family medicine physicians sometimes care for children in addition to adults.^20,30^ Primary language indicator identifies whether over 50% of the population with LEP speaks the same language. This highlights linguistic concentration, where a value of 1 indicates that a single language is spoken by a majority of the LEP population (over 50%), and 0 indicates that no single language is dominant.

### Statistical Analysis

Descriptive statistics were calculated as means with proportions, with survey weights. We tested for overall differences across categories using design-based *F* tests, and conducted post hoc pairwise comparisons for multiple comparisons across practice ownership categories.

We estimated logistic regression models to examine associations between practice-level language services availability and financial, ownership, and organizational capabilities, controlling for need-based factors and practice size. The outcome was whether or not the practice “always” delivered language services.

We estimated two models in order to examine the relationship between financial, ownership, and organizational capability factors associated with language services, incrementally adding covariates to assess potential confounders. Model 1 included payer mix variables only, which includes practice revenue from commercial insurance, Medicare, Medicaid, and self-pay/uninsured. Model 2 is fully adjusted with payer mix, ACO or capitation participation, and physician compensation from patient satisfaction or experiences, ownership, organizational capability variables, and language services need. Model estimates were converted to marginal effects and visualized using marginal effects plots. Sampling and non-response weights were applied. To assess multicollinearity, we calculated post-regression variance inflation factors (VIF), considering a threshold of 10 or higher as indicative of severe multicollinearity.^31^

We conducted sensitivity analyses to assess the robustness of our findings to different model specification decisions. First, we stratified the main regression model by low, medium, and high language services need to assess the consistency of associations across area-level patient needs. Second, we re-estimated the model using multilevel mixed-effects logistic regression to account for clustering at the county and state level. Finally, although the survey weights account for non-response probabilities, we re-estimated the descriptive analyses and regression results to assess the potential bias introduced by excluding responses.

All analyses were conducted using Stata, version 17 (StataCorp). Study procedures were reviewed and approved by the Committee for Protection of Human Subjects of the University of California, Berkeley. This cross-sectional study followed the Consensus Reporting of Survey Studies (CROSS) reporting guidelines.

## RESULTS

### Descriptive Analyses

Table 1 summarized weighted practice characteristics, stratified by language services availability. Language services were “always” provided in 54% of practices surveyed with the rest (46%) not consistently providing language services. Practices with language services differ on payer mix characteristics: they were more likely to have high Medicaid % revenue (> 20%) (32.5% vs 18.8%), and less likely to be have high commercial insurance revenue (> 50%) and high Medicare revenue (> 50%) (22.0% vs 33.6%, 10.3% vs 17.8%). In terms of ACO participation and capitated payments, and organizational capabilities, practices always offering language services were more likely to participate in an ACO or have capitated contracts (79.2% vs 69.6%), and training to care for immigrant and racially diverse patients (59.0% vs 36.9%), and were about as likely to have care processes for complex, high-need patients (62.8% vs 61.6%) compared to practices that did not. In terms of ownership, practices always providing language services were more likely to be owned by health systems (73.1% vs. 45.9%) and FQHCs (14.7% vs. 11.1%) than those that did not, but less likely to be owned by a medical group (3.7% vs 9.6%) or to be independent (8.5% vs 33.3%). Finally, a majority (57.9%) of practices were located in counties with low language services needs, 21% within moderate-need, and 22% within high-need counties. eFigure 1 illustrates a positive association between county-level language need and language-services availability. All differences were statistically significant at *p* ≤ 0.05. Post hoc pairwise comparisons (eTable 2) indicated that hospital or healthcare system owned practices reported higher availability of language services compared to Medical group owned practices (*p* ≤ 0.01).

### Regression Analyses

In Table 2, model 1, language services was positively and significantly associated with higher practice revenue from Medicaid (OR, 1.50 [95% CI 1.23, 1.83]; *p* ≤ 0.01), and lower practice revenue from commercial insurance (OR, 0.80 [95% CI 0.66, 0.96]; *p* ≤ 0.05) and uninsured/self-pay (OR, 0.70 [95% CI 0.53, 0.91]; *p* ≤ 0.01).

**Figure 1:**
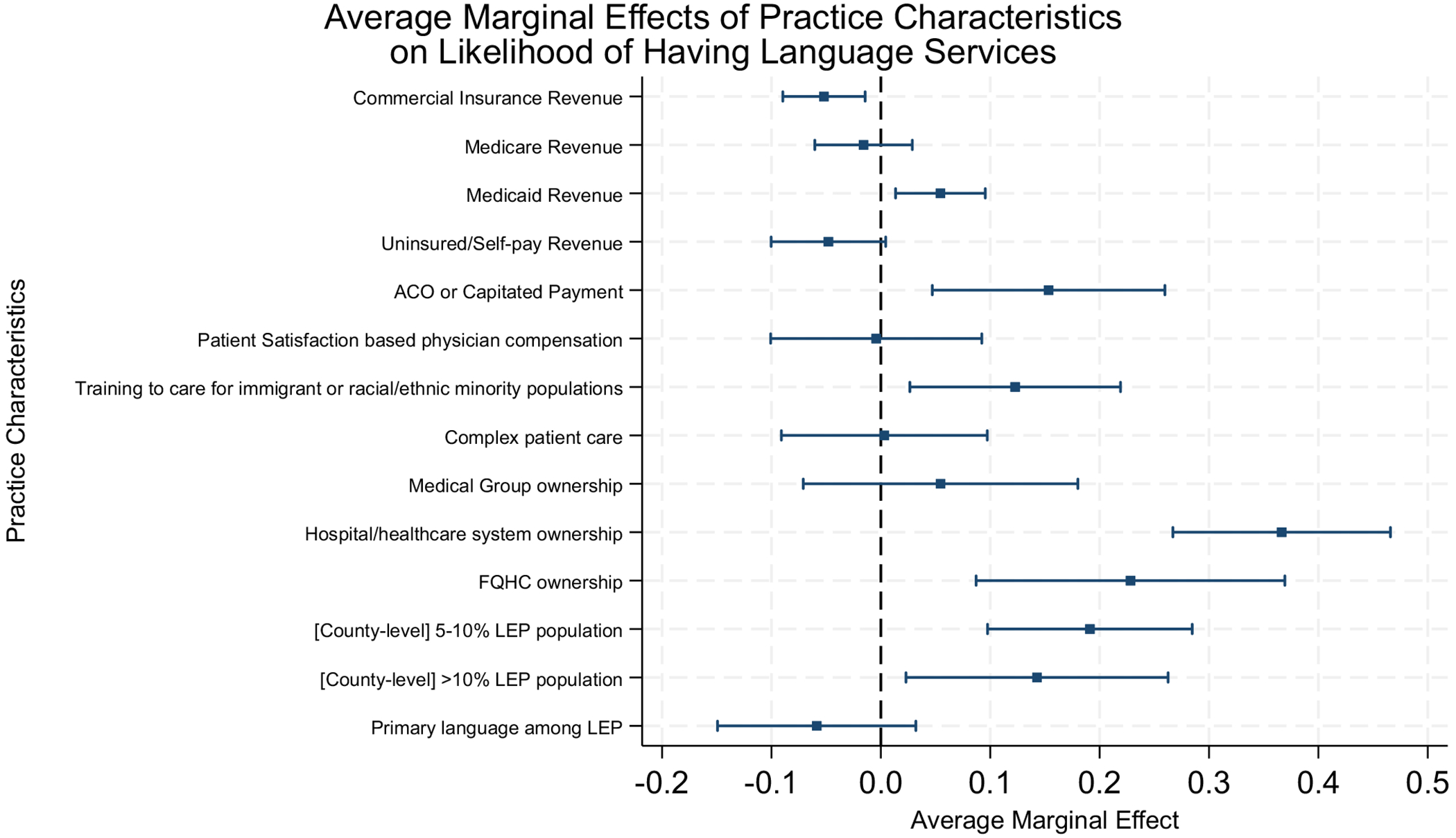
Average marginal effects of practice characteristics on likelihood of having language services. Blue squares represent marginal effects. Horizontal blue line denotes the 95% confidence interval (CI). Reference groups: ownership = independent; < 5% LEP population.

The fully adjusted model (Table 2, model 2) regression estimates were consistent with this relationship, with a higher share of revenue from Medicaid (OR, 1.33 [95% CI 1.07, 1.65]; *p* ≤ 0.01) but lower share of revenue from commercially insured patients (OR, 0.76 [95% CI 0.62, 0.93]; *p* ≤ 0.01), although the association with uninsured/self-pay was attenuated. Organizational characteristics associated with language services included participation in ACO or capitated payment (OR, 2.21 [95% CI 1.26, 3.87]; *p* ≤ 0.01), and training to care for immigrants and racially diverse patients (OR, 1.87 [95% CI 1.15, 3.05]; *p* ≤ 0.05). Practices were more likely to offer language services if owned by hospital or healthcare systems (OR, 6.01 [95% CI 3.63, 9.95]; *p* ≤ 0.01), or FQHCs (OR, 3.10 [95% CI 1.58, 6.08]; *p* ≤ 0.01) than those that were independently owned. To facilitate ease of interpretation of the fully adjusted model results, Fig. 1 depicts marginal mean values of language services associated with practice characteristics. Marginal effects values are provided in eTable 3 in the Appendix.

Variance inflation factor analyses indicated no severe multicollinearity among covariates (mean VIF = 3.44). Measures of commercial and Medicare revenue had slightly elevated VIF values (7.22 and 8.71, respectively), suggesting some correlation between these predictors. However, as these values remained below thresholds for severe multicollinearity (VIF ≥ 10), all variables were retained in the final model.

### Sensitivity Analyses

Results of sensitivity analyses that stratified analyses by language services need were consistent for practice ownership (Appendix, eTable 4). Training to care for immigrant and racially diverse patients, however, was only positively associated with language services among practices with low language services need. Similarly, ACO participation or capitated payment was only significant among low- and medium-need practices. Sensitivity analyses that accounted for clustering at the county- and state-level indicated that results remain unchanged (Appendix, eTable 5).

Our sensitivity analyses comparing excluded and included responses found similar practice characteristics based on missingness, although excluded practices (*n* = 18) reported lower levels of training to care for immigrant and diverse patients (mean 0.49 vs 0.73; *p* = 0.01) compared to non-missing practices.

## DISCUSSION

While language services are required by several federal laws,^6,10,12,13^ our findings indicate that they are not consistently provided in primary care practices and that payment incentives are significantly associated with the availability of language services. Practices that were more dependent on revenue from commercial insurance or self-paying patients were less likely to offer language services, while practices with greater reliance on Medicaid revenue were more likely. This could be because, unlike other payers, which generally lack standardized reimbursement for language services, Medicaid reimburses practices either directly or through their Medicaid managed care contracts.

Our findings also indicate that FQHCs and health care system-owned practices were more likely to always offer language services. This may be because larger organizations and systems have greater administrative capacity and staff resources to support the routine provision of language services, while FQHCs may have additional regulatory requirements and unique funding structures to support consistent language service provision. Additionally, training staff and clinicians to care for immigrants and racially diverse patients was also positively associated with availability of language services. This finding aligns with qualitative evidence citing challenges to effective language services such as collecting patient language preferences, understanding language services options, and training to use interpreters effectively.^14,32^ Care coordination challenges could potentially explain disparities in language services availability between large, networked physician practices and smaller independent practices.

When stratifying by language services need, we found no significant association between payer mix or ACO participation and language service availability among practices in high-need areas. One possible explanation for this is that practices in high-need communities may already have clinicians and staff who have language competencies of the patient population,^33^ thereby reducing their reliance on external language services. Additionally, these practices may need to provide language services as an essential part of patient care due to higher volume of LEP patients, regardless of payer mix or ACO participation. In contrast, practices in low- and moderate-need areas may face challenges in justifying investment in language services with inadequate payment, especially when a smaller proportion of their patient population require these services. In these areas, practices may be more reliant on ACO incentive payments to support language services or health plan provision of language services. Our measure does not identify the source of services; additional work should clarify whether provision is practice-based, centralized via independent practice associations or health plans, or vendor-provided.

To improve the routine availability of language services, policymakers and stakeholders should reassess how support for language services is incorporated into global payment rates to ensure that practices providing them are adequately compensated by all payers. Additionally, policymakers should consider developing policies that provide technical assistance to support independent physician practices in implementing and sustaining language services. Finally, the impact of innovative health technologies such as improved language preference data collection and artificial intelligence (AI)–enabled interpreter services should be examined. These innovations hold potential to improve language service availability, but implementation will require careful monitoring to ensure quality, access, and patient privacy.

### Limitations

There are some limitations that should be considered when interpreting this study’s results. First, we used a single survey question to assess language services availability. Other data, including electronic health record documentation of interpreter use, were not available to verify practice-reported use. Second, bilingual clinician capability and language concordant visit volume was not assessed, potentially limiting our understanding of language capabilities at some practices. Third, the availability of language services does not necessarily indicate the quality or modality of the services provided, and patient experience or outcomes. Finally, as financial factors and language availability were collected in a survey, we may find that the results reflect reporting and measurement bias and social desirability bias. Future research analyzing clinical and administrative data could enable understanding about how provision of professional medical interpretation by primary care practices impacts quality of care and patient outcomes.

## CONCLUSIONS

Higher Medicaid revenue, ACO participation and capitated payment, and FQHC and system ownership of practices are associated with language access, highlighting that policy reforms can influence the availability of language services at the practice-level. Aligning interpreter services resources and payment policies across payers may help primary care practices advance their efforts to improve quality of care for patients with LEP. Policymakers and policy advocates should monitor practice payments for interpreter services to ensure that all payers adequately support primary care practices in providing high-quality care to all patients, irrespective of their English language proficiency.

## Supplementary Material

Supplementary Material

The online version contains supplementary material available at https://doi.org/10.1007/s11606-025-09968-8.

## Figures and Tables

**Table 1:** Primary Care Physician Practice Characteristics, by Language Service Availability (Response Weighted)

	Overall *N*=1226	Always Available *N*= 616: (50.24%)	Not Always Available *N*=610 (49.76%)	*P*-value
Weighted N	10,738 (100.0%)	5,831 (54.3%)	4,907 (45.7%)	
**Payer Mix:**				0.004
High Commercial Insurance (>50%)	2,933 (27.3%)	1,283 (22.0%)	1,650 (33.6%)	
High Medicare (+Duals) (>50%)	1,472 (13.7%)	601 (10.3%)	872 (17.8%)	
High Medicaid (>20%)	2,821 (26.3%)	1,897 (32.5%)	924 (18.8%)	
High Uninsured/Self-pay (>20%)	563 (5.2%)	327 (5.6%)	236 (4.8%)	
No Majority Payer	2,949 (27.5%)	1,723 (29.6%)	1,226 (25.0%)	
ACO participation or capitated payment	8,032 (74.8%)	4,618 (79.2%)	3,414 (69.6%)	0.031
Physician compensation from Patient satisfaction or experiences	4,330 (40.3%)	2,445 (41.9%)	1,885 (38.4%)	0.524
Training to care for immigrant and racial/ethnic minority patients	5,255 (48.9%)	3,443 (59.0%)	1,812 (36.9%)	<0.001
Care processes for complex, high-need patients (≥50% scale)	6,685 (62.3%)	3,663 (62.8%)	3,022 (61.6%)	0.810
**Ownership:**				<0.001
Independently owned	2,131 (19.8%)	496 (8.5%)	1,635 (33.3%)	
Medical Group	690 (6.4%)	217 (3.7%)	473 (9.6%)	
A hospital or healthcare system	6,516 (60.7%)	4,262 (73.1%)	2,254 (45.9%)	
FQHC owned	1,401 (13.0%)	856 (14.7%)	545 (11.1%)	
Primary care physician count (mean, SD) (MD/DO)	10.17 (29.2)	11.86 (30.1)	8.15 (28.0)	0.102
Advanced practice clinician count (mean, SD)(PA, NP, CNS)	3.73 (5.2)	3.89 (5.7)	3.57 (5.4)	0.513
**Language Services Need (county-level):**				0.004
<5%	6,220 (57.9%)	2,952 (50.6%)	3,268 (66.6%)	
5–10%	2,211 (20.6%)	1,399 (24.0%)	812 (16.6%)	
>=10%	2,307 (21.5%)	1,480 (25.4%)	827 (16.9%)	
**Language diversity:**	6,153 (57.3%)	3,280 (56.3%)	2,873 (58.6%)	0.678
<50% of LEP population speak the same language				

Source: National Survey of Healthcare Organizations and Systems II (NSHOS II), IQVIA One Key Data, and American Community Survey (ACS)

*Abbreviations*: *ACO*, accountable care organization; *FQHC*, federally qualified health center; *MD*, Doctor of Medicine; *DO*, Doctor of Osteopathic Medicine; *PA*, physician assistant; *NP*, Nurse Practitioner; *CNS*, Clinical Nurse Specialist; *LEP*, limited English proficient

**Table 2:** Primary Care Physician Practice Characteristics Associated with Language Services in 2022

	Model 1 (Odds Ratios): Payer Mix Only		Model 2 (Odds Ratios): Adjusted	
**Practice patient care revenue from:**				
Commercial health insurance	0.80 [0.66, 0.96]	*	0.76 [0.62, 0.93]	**
Medicare (+ duals)	0.89 [0.72, 1.11]		0.92 [0.73, 1.16]	
Medicaid	1.50 [1.23, 1.83]	**	1.33 [1.07, 1.65]	**
Uninsured/Self-pay/Other	0.70 [0.53, 0.91]	**	0.78 [0.59, 1.03]	
ACO participation or capitated payment			2.21 [1.26, 3.87]	**
Physician compensation from Patient satisfaction or experiences			0.98 [0.59, 1.62]	
**Care Coordination:**				
Training to care for immigrant and racial/ethnic minority patients			1.87 [1.15, 3.05]	*
Care processes for complex, high-need patients			1.02 [0.62, 1.67]	
**Ownership: [Ref= Independent]**				
Medical Group			1.34 [0.69, 2.61]	
Hospital or healthcare system			6.01 [3.63, 9.95]	**
FQHC			3.10 [1.58, 6.08]	**
**Language Services Need (county-level):** [Ref= low]				
Moderate (5–10%)			2.75 [1.64, 4.60]	**
High (>10%)			2.10 [1.11, 3.97]	*
Primary language spoken among LEP			0.74 [0.46, 1.18]	
**Intercept**	2.21 [1.35, 3.62]	**	0.24 [0.10, 0.58]	**
**Number of observations**	1226		1226	

Source: National Survey of Healthcare Organizations and Systems II (NSHOS II), IQVIA One Key Data, and American Community Survey (ACS). Logistic regression was used to estimate the outcome variable. 95% confidence intervals are in brackets.

** *p* < 0.01,

* *p* < 0.05

*ACO* accountable care organization, *FQHC* federally qualified health center, *LEP* limited English proficient

## Data Availability

The authors do not have permission to share data.

## Abbreviations

ACO: Accountable care organization
FQHC: Federally qualified health center
LEP: Limited English proficient
