# Supplementary material for: U.S. Primary Care Practice Capabilities Linked to Language Services for Patients with Limited English Proficiency
Source: J Gen Intern Med. Author manuscript; Available in PMC 2025 Dec 10. (PMC12695077; doi:10.1007/s11606-025-09968-8)
Supplement: Supplementary Material [file NIHMS2126683-supplement-Supplementary_Material.docx]

**U.S. Primary Care Practice Capabilities Linked to Language Services for Patients with Limited English Proficiency**

Supplementary File

[eFigure 1: Percent of Limited English Proficient speakers in county and Language Services Availability 2](#_f7nmt0y3dx52)

[eTable 1: Correlations of Composite Measures 2](#_el3fvxcrus50)

[eTable 2: Post-Hoc Analysis: Pairwise Comparisons of Ownership Categories on the Provision of Language Services 4](#_g5tyvp3eb36g)

[eTable 3: Primary Care Physician Practice Characteristics Associated with Language Services- Marginal Effects 5](#_9quflkhh2arx)

[eTable 5: Primary Care Physician Practice Characteristics Associated with Language Services with County and State-level clustering. 8](#_x2s9y2mygn8r)

**Appendix**:

# eFigure 1: Percent of Limited English Proficient speakers in county and Language Services Availability


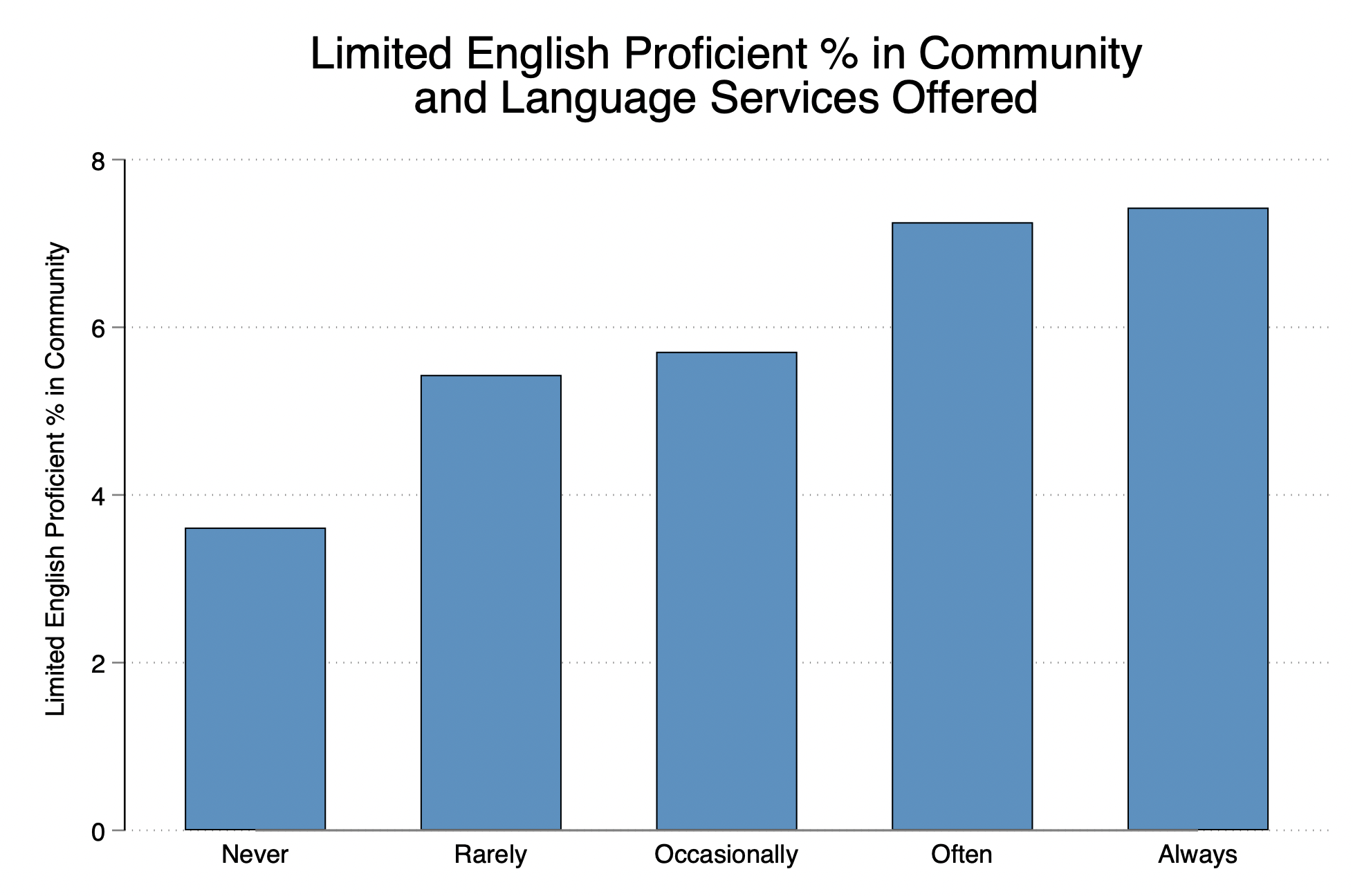


# eTable 1: Correlations of Composite Measures

| **Measure** | **Complex patient care** | **Training scale** |
| --- | --- | --- |
| **Complex patient care** | 1.00 | 0.21*** |
| **Training scale** | 0.21*** | 1.00 |

*Note: Pearson correlations with p values. p<0.05 = *, p<0.001 = **. None of the scales are highly correlated (all r<0.70).

# eTable 2: Post-Hoc Analysis: Pairwise Comparisons of Ownership Categories on the Provision of Language Services

| **Contrast** | **Difference in Marginal Prediction** | **Std. Error** | **z** | **p** | **95% CI** |
| --- | --- | --- | --- | --- | --- |
| Medical Group vs Independent | 0.29 | 0.34 | 0.87 | 0.95 | –0.60, 1.19 |
| Hospital/health system vs Independent | **1.79** | 0.26 | 6.98 | **<0.001** | 1.12, 2.47 |
| FQHC vs Independent | **1.13** | 0.34 | 3.29 | **0.01** | 0.23, 2.04 |
| Hospital/health system vs Medical Group | **1.50** | 0.35 | 4.33 | **<0.001** | 0.59, 2.41 |
| FQHC vs Medical Group | 0.84 | 0.41 | 2.03 | 0.23 | –0.25, 1.92 |
| FQHC vs Hospital/health system | –0.66 | 0.32 | –2.10 | 0.20 | –1.49, 0.17 |

#

| Source: National Survey of Healthcare Organizations and Systems II (NSHOS II), IQVIA One Key Data, and American Community Survey (ACS). Estimates are differences in marginal linear predictions from survey-weighted model 2 with Sidak adjustment for multiple comparisons. Statistically significant results at *p*≤0.05 are shown in **bold**.  Abbreviations: FQHC, federally qualified health center. | | | |
| --- | --- | --- | --- |

# eTable 3: Primary Care Physician Practice Characteristics Associated with Language Services- Marginal Effects

| **Practice patient care revenue from:** |  |  |
| --- | --- | --- |
| Commercial health insurance | 0.76 [0.62, 0.93] | ** |
| Medicare (+ duals) | 0.92 [0.73, 1.16] |  |
| Medicaid | 1.33 [1.07, 1.65] | ** |
| Uninsured/Self-pay/Other | 0.78 [0.59, 1.03] |  |
| ACO participation or capitated payment | 2.21 [1.26, 3.87] | ** |
| Physician compensation from  Patient satisfaction or experiences | 0.98 [0.59, 1.62] |  |
| **Organizational Capabilities:**  Training to care for immigrant and racially diverse patients | 1.87 [1.15, 3.05] | * |
| Care processes for complex, high-need patients | 1.02 [0.62, 1.67] |  |
| **Ownership:**  [Ref= Independent] | 1.34 [0.69, 2.61] |  |
| Medical Group | 6.01 [3.63, 9.95] |  |
| Hospital or healthcare system | 3.10 [1.58, 6.08] | ** |
| FQHC | 2.75 [1.64, 4.60] | ** |
|  | 2.10 [1.11, 3.97] |  |
|  | 0.74 [0.46, 1.18] |  |
| **Limited English Proficiency: (county-level):** [Ref= low] | 0.24 [0.10, 0.58] |  |
| Moderate (5-10%) | 0.76 [0.62, 0.93] | ** |
| High (≥10%) | 0.92 [0.73, 1.16] | * |
| Primary language spoken among LEP | 1.33 [1.07, 1.65] |  |
| **Intercept** | 0.78 [0.59, 1.03] | ** |
| **Number of observations** | 1226 |  |

Source: National Survey of Healthcare Organizations and Systems II (NSHOS II), IQVIA One Key Data, and American Community Survey (ACS). Logistic regression was used to estimate the outcome variable. 95% Confidence intervals are in brackets. ** p<.01, * p<.05

Abbreviations: ACO, accountable care organization; FQHC, federally qualified health center; LEP, limited English proficient.

eTable 4: Primary Care Physician Practice Characteristics Associated with Language Services by Language Services Need

|  | | | | | | |
| --- | --- | --- | --- | --- | --- | --- |
|  | Low Need | | Moderate Need | | High Need | |
| **Practice patient care revenue from:** |  |  |  |  |  |  |
| Commercial health insurance | 0.69 [0.52, 0.93] | * | 0.71 [0.50, 1.00] |  | 0.86 [0.59, 1.26] |  |
| Medicare (+ duals) | 1.03 [0.74, 1.44] |  | 0.56 [0.38, 0.81] | ** | 0.98 [0.69, 1.39] |  |
| Medicaid | 1.47 [1.04, 2.08] | * | 2.10 [1.38, 3.20] | ** | 1.09 [0.83, 1.44] |  |
| Uninsured / Self-pay / Other | 0.64 [0.39, 1.06] |  | 0.88 [0.58, 1.33] |  | 0.97 [0.58, 1.62] |  |
| ACO participation or capitated payment | 2.52 [1.21, 5.23] | * | 3.59 [1.33, 9.67] | * | 1.01 [0.38, 2.70] |  |
| **Physician compensation from:** |  |  |  |  |  |  |
| Patient satisfaction/experiences | 0.93 [0.48, 1.82] |  | 1.81 [0.69, 4.71] |  | 0.78 [0.26, 2.37] |  |
| **Organizational Capabilities:** |  |  |  |  |  |  |
| Training to care for immigrant/diverse patients | 2.27 [1.18, 4.34] | * | 1.84 [0.68, 4.98] |  | 1.48 [0.49, 4.44] |  |
| Care processes for complex, high-need patients | 0.82 [0.42, 1.63] |  | 0.97 [0.39, 2.39] |  | 1.38 [0.52, 3.71] |  |
| **Ownership (Ref = Independent):** |  |  |  |  |  |  |
| Medical Group | 1.07 [0.36, 3.18] |  | 1.73 [0.53, 5.70] |  | 2.89 [0.98, 8.58] |  |
| Hospital/healthcare system | 4.48 [2.35, 8.56] | ** | 11.66 [4.46, 30.51] | ** | 8.27 [2.70, 25.29] | ** |
| FQHC | 2.77 [1.06, 7.27] | * | 1.31 [0.34, 5.12] |  | 4.14 [1.36, 12.62] | * |
| Primary language spoken among LEP | 0.58 [0.31, 1.07] |  | 1.08 [0.44, 2.66] |  | 0.78 [0.34, 1.79] |  |
| Intercept | 0.33 [0.11, 1.03] |  | 0.41 [0.07, 2.49] |  | 0.48 [0.08, 2.77] | ** |
| **Number of observations** | 654 |  | 271 |  | 301 |  |

| Source: National Survey of Healthcare Organizations and Systems II (NSHOS II), IQVIA One Key Data, and American Community Survey (ACS). Logistic regression was used to estimate the outcome variable. 95% Confidence intervals are in brackets. ** p<.01, * p<.05  Abbreviations: ACO, accountable care organization; FQHC, federally qualified health center; LEP, limited English proficient. | | | | | | |
| --- | --- | --- | --- | --- | --- | --- |

We then performed a three-level hierarchical multivariable random intercept regression model to examine potential clustering effects at the county and state level. Based on the likelihood ratio (LR) chi2 test, the difference in fit statistics between the fully adjusted model and base model was not statistically significant.

# eTable 5: Primary Care Physician Practice Characteristics Associated with Language Services with County and State-level clustering.

| All Models | | | | |
| --- | --- | --- | --- | --- |
|  | Language Services (VCM) |  | Language Services  (Adjusted) | |
| **Practice patient care revenue from:** |  |  |  |  |
| Commercial health insurance |  |  | 0.96 [0.85, 1.08] |  |
| Medicare (+ duals) |  |  | 0.82 [0.71, 0.94] | ** |
| Medicaid |  |  | 1.33 [1.16, 1.52] | ** |
| Uninsured / Self-pay / Other |  |  | 0.83 [0.69, 1.01] |  |
| ACO participation or capitated payment | – | – | 1.63 [1.19, 2.22] | ** |
| Physician compensation from patient satisfaction/experiences | – | – | 1.04 [1.01, 1.07] | * |
| **Organizational Capabilities:** |  |  |  |  |
| High training to care for immigrant/diverse patients | – | – | 1.91 [1.45, 2.51] | ** |
| High care processes for complex, high-need patients | – | – | 1.03 [0.79, 1.36] |  |
| **Ownership (ref = independent):** |  |  |  |  |
| Medical Group | – | – | 2.14 [1.31, 3.49] | ** |
| Hospital or healthcare system | – | – | 5.33 [3.70, 7.68] | ** |
| FQHC | – | – | 3.98 [2.44, 6.51] | ** |
| **Limited English Proficiency (county-level, ref = low):** |  |  |  |  |
| Moderate (5–10%) | – | – | 1.48 [1.05, 2.08] | * |
| High (≥10%) | – | – | 1.46 [1.00, 2.15] |  |
| Primary language spoken among LEP | – | – | 1.00 [0.75, 1.33] |  |
| **Intercept** | 0.92 [0.76, 1.12] |  | 0.19 [0.11, 0.34] | ** |
| State-level cluster | 0.21 [0.09, 0.51] |  | 0.04 [0.00, 0.63] |  |
| County cluster within state | 0.29 [0.08, 1.07] |  | 0.10 [0.00, 6.77] |  |
| **Number of observations** | 1,226 |  | 1,226 |  |
| Source: National Survey of Healthcare Organizations and Systems II (NSHOS II), IQVIA One Key Data, and American Community Survey (ACS). Logistic regression was used to estimate the outcome variable. 95% Confidence intervals are in brackets. ** p<.01, * p<.05. Note: Estimates are transformed only in the first equation to odds ratios. Likelihood Ratio (LR) test vs. logistic model: chi2(2) = 0.91, Prob > chi2 = 0.6352  Abbreviations: ACO, accountable care organization; FQHC, federally qualified health center; LEP, limited English proficient. | | | | |
